# Multimodal modeling of emotion regulation in interactive art therapy: EEG and PANAS-based dynamic simulation

**DOI:** 10.3389/fpsyg.2025.1665506

**Published:** 2025-12-01

**Authors:** Xiaowei Chen, Miaomiao Zeng

**Affiliations:** 1 College of Arts, Zhejiang Shuren University, Hangzhou, China; 2 School of Education, Zhaoqing University, Zhaoqing, China

**Keywords:** emotion regulation, interactive art therapy, EEG, affective shift, PANAS, nonlinear simulation, dynamic simulation, feedback adaptation

## Abstract

**Introduction:**

Emotion regulation within immersive art therapy emerges from the complex interplay of cognitive control, physiological arousal, and affective appraisal. Although this relationship has theoretical significance, the predictive connections between neurophysiological markers and subjective affective dynamics have yet to be thoroughly investigated.

**Methods:**

This study proposes a dual-model framework that integrates linear regression and nonlinear simulation to examine how electroencephalographic (EEG) features and PANAS-based self-reports jointly predict emotional change. EEG and affective data were collected from 50 participants following exposure to an interactive installation. Three predictors were derived: Theta_Change (cognitive load), Gamma_Change (physiological arousal), and Affective_Shift (subjective valence change).

**Results:**

Bootstrapped regression analysis (*n* = 1,000) identified Affective_Shift as the most robust predictor of both positive affect change (Δ_Positive: *β* = 1.69, 95% CI [−0.53, 3.90]) and negative affect change (Δ_Negative: β = −3.96, 95% CI [−5.84, −2.16]). Gamma_Change also contributed significantly to positive emotional outcomes, while Theta_Change exhibited nonlinear effects contingent on initial affective states. Dynamic simulations conceptually illustrated stable emotional payoff trajectories and adaptive EEG shifts, offering an exploratory model of feedback-sensitive affective regulation.

**Discussion:**

Together, these findings support a multidimensional model of emotion regulation that integrates subjective evaluation with neurophysiological indicators. The results are consistent with Gross’s process model, Russell’s circumplex theory, and Kuppens’s emotion dynamics framework. The proposed computational approach provides a mechanistic understanding and actionable insights for designing affect-aware, adaptive environments in therapeutic and artistic domains.

## 1 Introduction

Although interactive art has garnered increasing attention as a means of emotional engagement, a nuanced understanding of how such experiences distinctly facilitate the enhancement of positive affect and the attenuation of negative affect remains underdeveloped. This gap is particularly salient at the neurophysiological level, where the dynamic mechanisms underlying emotional transitions have yet to be systematically elucidated. While creative therapies are increasingly embraced as non-pharmacological interventions for emotion regulation, empirical evidence detailing their neurodynamic foundations—especially within feedback-sensitive and interactive environments—remains limited.

Prior research has demonstrated that engaging in artistic activities can elicit both immediate emotional relief and long-term psychological integration (Kaimal et al., 2020; Stuckey and Nobel, 2010), with corresponding neural activity observed in frontal and parietal EEG regions (Belkofer et al., 2014). However, such findings predominantly rely on subjective self-report measures or static pre/post comparisons, providing only a fragmented view of the temporal unfolding of emotional responses. What remains absent is a process-oriented framework that captures how neurophysiological markers co-evolve with affective experience during immersive, participatory interactions.

Moreover, existing studies often examine EEG and self-report data in isolation, thereby overlooking the synergistic potential of multimodal integration. Although theoretical constructs such as the Expressive Therapies Continuum (ETC) and empirical evidence on frontal alpha asymmetry indicate modality-specific emotional responses (Hinz, 2020; Harmon-Jones and Gable, 2018), the lack of dynamic modeling inhibits our understanding of how individuals adaptively regulate emotion in real-time (Gross, 2015). Consequently, despite the expanding interest in creative engagement for emotion regulation, current approaches remain limited by static, often black-box models that fail to capture feedback-driven emotional modulation.

To address these limitations, the present study proposes a dual-pathway modeling framework that integrates EEG-derived features—Theta_Change (representing cognitive load) and Gamma_Change (indicating physiological arousal)—with PANAS-based measures of affective change. By combining linear regression with gradient-based nonlinear simulation, we aim to model the co-adaptive interplay between neurophysiological activity and self-reported affective states, ultimately identifying the input configurations that promote optimal emotional outcomes in interactive art contexts. Specifically, we explore the following research questions:

- To what extent do Theta_Change and Gamma_Change predict positive and negative affect changes?
- Does Affective_Shift mediate this relationship?
- Can a game-theoretic simulation identify optimal emotion regulation strategies across the neuro-affective space?
- Do multimodal models outperform EEG-only or PANAS-only approaches in predictive accuracy?

These inquiries are grounded in three complementary theoretical frameworks: Gross’s Process Model of Emotion Regulation, Russell’s Circumplex Model of Affect, and Kuppens and Verduyn’s Emotion Dynamics Theory. Together, these models conceptualize affective feedback as a dynamic, cognitively modulated, and context-sensitive process, offering a robust theoretical lens for understanding emotional adaptation within immersive artistic environments.

## 2 Literature review

The intersection of neuroscience and creative therapies has attracted increasing scholarly attention, particularly regarding the neurophysiological mechanisms underlying emotional regulation in art-based interventions. Growing empirical evidence suggests that artistic media, task structures, and sensory immersion can dynamically modulate emotional and cognitive states. For example, clay-based and drawing activities activate distinct but overlapping cortical networks involved in sensorimotor integration and affect regulation (Lusebrink and Hinz, 2020; Barnett and Vasiu, 2024). In a related study, Wang et al. (2025) demonstrated that an immersive VR-based mindfulness intervention could influence stress reactivity and attentional regulation, as evidenced by EEG-derived relaxation metrics. However, these findings predominantly rely on static pre/post comparisons or unimodal analyses, thus limiting the capacity to capture the temporal evolution of emotional processes.

Recent advances in affective neuroscience and computational modeling have addressed these limitations by providing tools to examine emotion as a dynamic and multidimensional phenomenon. Research has shown that theta and gamma band oscillations are associated with cognitive load and physiological arousal, respectively, and that fluctuations in these frequencies can reflect moment-to-moment emotional states (Balconi et al., 2025; Koval et al., 2023). In immersive settings—such as virtual reality (VR) environments or interactive installations—combining EEG measures with self-report instruments like the Positive and Negative Affect Schedule (PANAS) enables a more fine-grained understanding of affective transitions (Wang et al., 2025). Nonetheless, many existing approaches maintain a signal-centric perspective, often overlooking the integrative role of subjective experience, contextual factors, and regulatory intent in shaping emotional responses.

Recent theoretical models emphasize the process-oriented and dynamic nature of emotion regulation to interpret emotional transitions more comprehensively. Gross’s Process Model (2015) delineates four sequential stages—situation selection, attentional deployment, cognitive change, and response modulation—offering a conceptual framework for linking neural indicators (e.g., theta/gamma activity) with regulatory behaviors. Similarly, Russell’s Circumplex Model of Affect (1980) provides a valence-arousal mapping space that aligns with EEG-derived features. Kuppens and Verduyn (2017) emphasize intra-individual variability and the temporal dynamics of emotional experience. Together, these frameworks underscore the need for analytic approaches that capture both affective states and the feedback-sensitive mechanisms by which individuals adapt their regulatory strategies over time.

In applied settings—particularly within therapeutic and interactive art contexts—adaptive systems utilizing EEG-driven feedback loops have been developed to enhance user engagement and emotional benefit (Koval et al., 2023; Jiang et al., 2025). However, such systems primarily emphasize real-time affective responsiveness, often without accounting for the longer-term trajectories of emotional regulation. Recent advances in affective neuroscience and computational design have emphasized emotion regulation’s dynamic, goal-directed nature. Instead of treating affect as a static state, scholars have proposed trajectory-based models (Koval et al., 2023; Southward et al., 2021) in which individuals navigate emotion regulation landscapes toward desired affective outcomes—guided by cognitive control, physiological arousal, and regulatory intent (Slovak et al., 2023).

Despite these promising developments, several critical gaps persist. First, few empirical models treat emotion regulation as a co-evolving system involving cognitive load, physiological arousal, and affective intent. Second, even fewer studies integrate EEG and PANAS data within a temporally dynamic and feedback-sensitive modeling framework. Third, the inherently adaptive and strategic nature of emotion regulation—how individuals adjust tactics in response to shifting internal states—has not been sufficiently captured, especially within arts-based therapeutic environments.

To address these challenges, the present study proposes a multimodal simulation framework that combines EEG and PANAS features to model affective outcomes in interactive art environments. Specifically, we define three key predictors: Theta_Change (cognitive load), Gamma_Change (physiological arousal), and Affective_Shift (emotional change), and assess their influence on two emotion regulation outcomes: Delta_Positive_Delta and Delta_Negative_Delta. A linear ridge regression model estimates global affective trends, while a nonlinear optimization model simulates emotional payoff surfaces across the cognitive-affective landscape. This integrative approach aims to identify optimal neuro-affective configurations and support interpretable, feedback-driven emotion regulation strategies in immersive art therapy settings.

## 3 Research design

### 3.1 Theoretical framework

**Figure 1:**
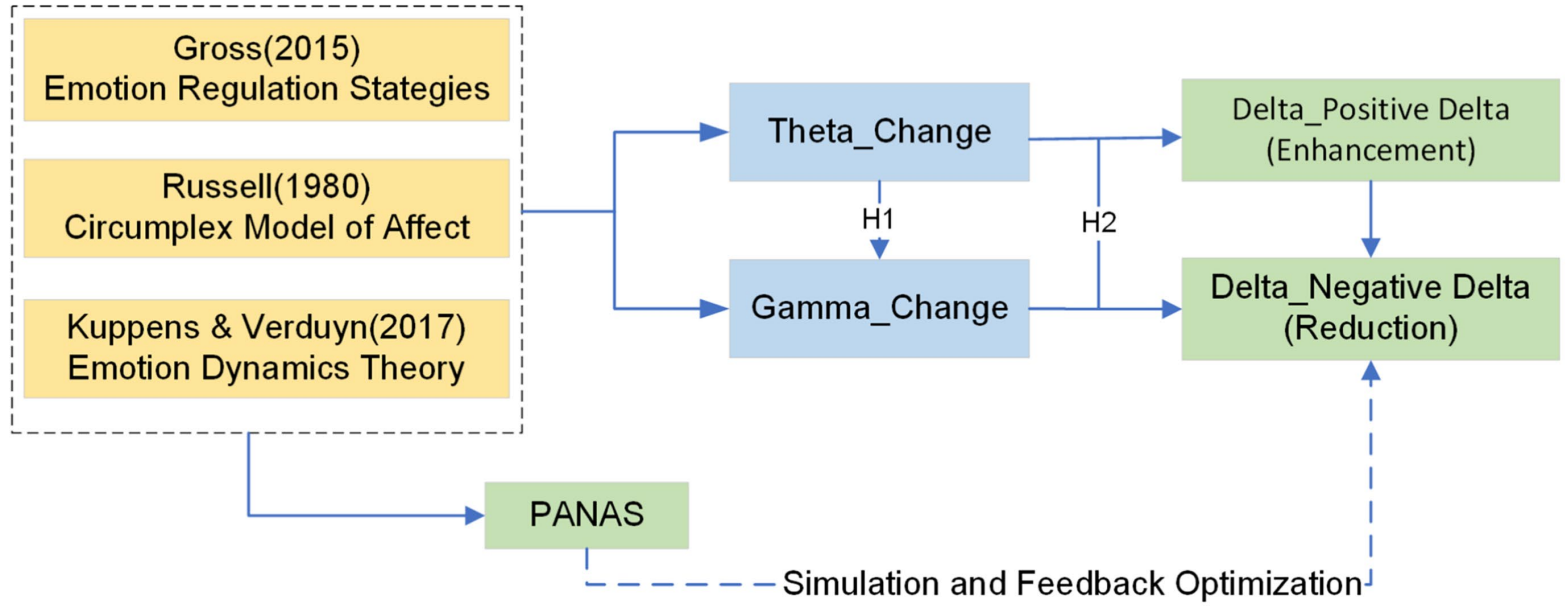
Theoretical framework and hypothesis structure.

To model emotion regulation in the context of interactive artmaking, this study synthesizes three influential theoretical perspectives from affective science: (1) Gross’s Process Model of Emotion Regulation, (2) Russell’s Circumplex Model of Affect, and (3) Kuppens’s Emotion Dynamics Theory, as illustrated in Figure 1. These models offer a psychologically grounded and computationally adaptable foundation for interpreting EEG and PANAS signals across cognitive, affective, and temporal dimensions.

Gross’s Process Model (Gross, 2015) conceptualizes emotion regulation as a multistage process comprising situation selection, attentional deployment, cognitive reappraisal, and response modulation. In the current study, these stages are operationalized through distinct neurophysiological indicators: Theta_Change (4–8 Hz), commonly associated with attentional control and working memory demands, is treated as an index of cognitive load, whereas Gamma_Change (30–50 Hz), linked to sensory integration and affective intensity, serves as a marker of physiological arousal. The PANAS-derived Affective_Shift variable captures the perceived emotional change, representing the subjective outcome of the regulation process as experienced by participants.

Russell’s Circumplex Model of Affect (Russell, 1980), by contrast, does not describe regulation mechanisms. Instead, it provides a structural mapping of affective experience along two orthogonal dimensions: valence (pleasant–unpleasant) and arousal (high–low activation). In this study, we use the circumplex primarily as a *spatial framework* to situate PANAS-derived affective changes and EEG correlates within a common affective space, rather than as a theory of regulation itself.

Kuppens’s Emotion Dynamics Theory (Kuppens and Verduyn, 2017; Koval et al., 2023) emphasizes the temporal unfolding and adaptive variability of emotional experience, portraying affective states as dynamic trajectories shaped by the interplay between internal goals and external feedback. Rather than treating emotions as fixed endpoints, this perspective views them as emergent from ongoing self-regulatory processes. Accordingly, our modeling strategy integrates linear regression to capture global affective patterns and nonlinear simulations to examine feedback-sensitive emotional dynamics. This theoretical lens also supports our interpretation of PANAS and EEG-derived deltas as temporal indicators of regulation in action.

Taken together, these three perspectives serve complementary roles: Gross informs the mechanistic process of regulation, Russell offers a structural space for mapping affect, and Kuppens highlights the temporal dynamics of adaptation. Our framework does not treat them as a single unified model but as distinct lenses that jointly guide the interpretation of EEG and PANAS data.

### 3.2 Hypothesis

Grounded in an integrated theoretical framework drawing from Gross’s emotion regulation theory, Russell’s circumplex model of affect, and Kuppens and Verduyn’s theory of emotion dynamics, this study proposes a series of hypotheses to explore how neurophysiological indicators and self-reported affective responses interact to shape emotional outcomes in the context of interactive art therapy. This framework conceptualizes emotion regulation as a feedback-sensitive process involving cognitive effort, physiological arousal, and perceived shifts in emotional valence. The study adopts a dual modeling approach incorporating linear regression and nonlinear simulation techniques to examine global tendencies and individual regulatory dynamics.

*H1*: Cognitive load modulates negative emotion regulation.

Theta_Change, an index of frontal-midline theta activity, is interpreted as a neural correlate of cognitive control and attentional engagement. Drawing on principles from cognitive load theory and Gross’s multistage model, we hypothesize that moderate increases in Theta_Change will facilitate the downregulation of negative emotion (Δ_Negative_Delta). Conversely, insufficient or excessive cognitive effort may disrupt effective regulation, leading to suboptimal emotional outcomes.

*H2*: Physiological arousal contributes to the enhancement of positive emotion.

Gamma_Change, characterized by high-frequency EEG oscillations, serves as an indicator of physiological arousal and affective salience. In line with arousal-based models and Russell’s valence–arousal framework, we hypothesize that elevated Gamma_Change will be associated with increased positive affect (Δ_Positive_Delta). However, extreme arousal levels may yield diminishing returns or even counterproductive effects, reflecting nonlinearities in emotional responsiveness.

*H3*: Multimodal models integrating both EEG and PANAS data will outperform unimodal models (EEG-only or PANAS-only) in predicting emotional change.

This hypothesis aligns with prior findings suggesting that combining objective physiological measures with subjective affective reports enhances predictive accuracy and interpretability in affective computing contexts. The comparative performance will be assessed through model evaluation metrics (e.g., RMSE, R^2^) across different input configurations.

### 3.3 Empirical analytical framework

This section outlines the data sources, variable operationalization, and analytical strategies for testing the proposed hypotheses. A dual-model analytical framework, integrating linear regression and nonlinear optimization techniques, was adopted to capture broad behavioral patterns alongside localized, feedback-sensitive emotional dynamics.

#### 3.3.1 Dataset and participants

This study employed a publicly available dataset titled *Harnessing New Frontiers in Art Therapy: An Interactive Installation Integrating EEG, VR, and AIGC for the Alleviation of Negative Emotions* (Chenghong et al., 2024), hosted on the Science Data Bank (DOI: 10.57760/sciencedb.08758). The dataset comprises multimodal recordings, including electroencephalographic (EEG) signals and self-reported affective responses, collected from 50 university students (27 females, 23 males; *M* = 21.00 years, *SD* = 1.89) who engaged with an immersive digital art installation designed to evoke affective responses. Participants completed the Positive and Negative Affect Schedule (PANAS) immediately before and after the installation session, providing subjective measures of affective change. Simultaneously, EEG signals were captured using a 32-channel Emotiv Flex wireless headset operating at a sampling rate of 128 Hz. Participants with a history of neurological disorders or psychoactive substance use were excluded from the original dataset.

While this dataset offers valuable insights into emotion regulation in ecologically valid, art-based contexts, several methodological limitations warrant consideration. First, the sample comprises exclusively non-clinical university students, which may limit the generalizability of findings to other demographic or clinical populations. Second, emotional elicitation was achieved via a multisensory interactive installation rather than standardized laboratory stimuli, thus prioritizing ecological validity over experimental control. Third, affective dynamics were assessed using only two measurement points (pre- and post-intervention PANAS scores), limiting the temporal granularity of emotional change analysis. Similarly, EEG features were derived from aggregated spectral power estimates (e.g., Theta_Change and Gamma_Change) rather than whole time series or event-related potentials. Although these design choices enable theoretically interpretable modeling, they also indicate opportunities for more temporally nuanced analyses in future research.

#### 3.3.2 EEG preprocessing and feature extraction

EEG preprocessing followed established affective neuroscience protocols (Hosseini et al., 2023). A fourth-order Butterworth band-pass filter (1–50 Hz) was applied to remove low-frequency drift and high-frequency noise. Independent Component Analysis (ICA) was conducted to eliminate ocular and muscular artifacts. Cleaned EEG data were then segmented into 2-s non-overlapping epochs, followed by baseline correction to enhance inter-epoch comparability and reduce variability due to pre-stimulus fluctuations.

Feature extraction was done across three computational domains: time-domain, frequency-domain, and time-frequency representations. Time-domain features included statistical descriptors such as mean, standard deviation, skewness, and kurtosis. Frequency-domain features were computed using power spectral density (PSD) estimates derived from Fast Fourier Transform (FFT), covering canonical EEG bands: delta (0.5–4 Hz), theta (4–8 Hz), alpha (8–13 Hz), beta (13–30 Hz), and Gamma (>30 Hz) (Algarni et al., 2022). Time-frequency features were extracted via Short-Time Fourier Transform (STFT) and Continuous Wavelet Transform (CWT) to capture evolving transient oscillatory patterns.

Among the extracted features, frontal midline theta and parietal Gamma were selected as primary predictors based on their well-documented roles in cognitive control and physiological arousal during emotion regulation (Balconi et al., 2025; Kuppens and Verduyn (2017); Jiang et al., 2021). These components were aggregated as Theta_Change and Gamma_Change, representing pre/post changes in band-specific power. Their inclusion was further supported by empirical evidence demonstrating their sensitivity to emotionally evocative, interactive, and art-based tasks (Dubey et al., 2024; Tang et al., 2022). While features from all three computational domains were initially extracted, only frequency-domain and time-frequency features were retained for downstream analysis, owing to the non-Gaussian distribution of the data and the study’s focus on oscillatory dynamics associated with effective transitions. EEG change scores (Theta_Change and Gamma_Change) were computed as raw pre–post differences and were not standardized (e.g., z-scored or log-transformed) prior to regression analysis.

#### 3.3.3 Statistical analysis

Normality checks using the Shapiro–Wilk test revealed significant deviations from the Gaussian distribution in EEG features and PANAS scores. Consequently, non-parametric statistical tests were employed to ensure analytical robustness under non-normal conditions. Table 1 summarizes the tests and their purposes, including the Mann–Whitney U test, Kruskal–Wallis H test, Spearman’s rank correlation, Cliff’s delta for effect size, and bootstrapped confidence intervals (*n* = 1,000). This decision is consistent with the exploratory nature of the study and the relatively small sample size (*n* = 50)—a limitation commonly encountered in affective neuroscience research involving immersive experimental paradigms (Wang et al., 2025). To enhance the validity of inferential results, bootstrapped confidence intervals (1,000 resamples) were computed for all regression coefficients. This approach reduces reliance on parametric assumptions and aligns with best practices in small-sample, nonlinear modeling contexts (Dogan, 2007).

Although the dataset adopts a pre/post structure, recent simulation-based studies demonstrate that dynamic modeling remains feasible even with discretized time points, provided a feedback-sensitive framework is adopted (Schauberger and Tutz, 2023). Following this rationale, the current study constructs a dynamic response surface (emotional payoff landscape) to simulate adaptive strategy adjustments across the affective space. This modeling approach transforms static pre/post observations into interpretable trajectories of affective change under contextual constraints.

**Table 1:** Summary of non-parametric statistical tests employed.

Statistical test	Purpose
Mann–Whitney U test	To evaluate differences in EEG features before and after installation.
Kruskal–Wallis H test	To compare EEG band contributions across multiple frequency ranges.
Spearman’s rank correlation	To assess monotonic relationships between EEG activity and PANAS scores.
Cliff’s Delta	To estimate effect sizes in small-sample, non-parametric contexts.
Bootstrapped CI (*n* = 1,000)	To compute robust confidence intervals for model coefficients.

Bootstrapped estimates of central tendency and 95% confidence intervals for key variables are presented in Table 2. While Delta_Negative showed a statistically significant reduction, the observed increase in Delta_Positive did not reach significance, as its confidence interval included zero.

**Table 2:** Bootstrap of estimates of key variables.

Variable	Mean	95% CI Lower	95% CI Upper
Theta_Change	2.67218E-11	−3.09131E-12	7.30581E-11
Gamma_Change	1.66029E-12	5.51989E-13	3.06104E-12
Delta_Positive	1.651578947	−0.631578947	4.052631579
Delta_Negative	−3.925368421	−5.894736842	−2.052631579

### 3.4 Analytical strategy: dual-model approach to emotion regulation modeling

The dual-model analytical framework is theoretically grounded in Gross’s Process Model of Emotion Regulation and Kuppens’s Theory of Emotion Dynamics. The linear model corresponds to Gross’s modulation stage, capturing general associations and interaction effects among cognitive, physiological, and affective variables. In contrast, the nonlinear simulation model reflects the feedback-sensitive and adaptive nature of emotion regulation, as proposed by Kuppens, modeling emotional change as a convergence process over time.

#### 3.4.1 Variable construction

To capture key components of emotion regulation, three predictor variables were defined:

- Theta_Change: Change in frontal-midline theta (4–8 Hz), indexing cognitive control (Balconi et al., 2025).
- Gamma_Change: Change in gamma band (30–50 Hz) in frontal–parietal regions, reflecting arousal and readiness (Lahijanian and Aghajan, 2023).
- Affective_Shift: Net change in PANAS scores, representing subjective reappraisal (Russell, 1980; Kuppens and Verduyn, 2017).

Two outcome variables—Delta_Positive and Delta_Negative—were computed as the differences between pre-and post-session PANAS scores.

#### 3.4.2 Linear regression model

A multivariate linear regression model was constructed to estimate both the direct and interaction effects:


$$\begin{array}{l} \mathrm{\Delta E}=\beta_{0}+\beta_{1}*\Delta \theta +\beta_{2}*\Delta \gamma +\beta_{3}*\mathrm{AS}+\\ \beta_{4}*(\Delta \theta *\mathrm{AS})+\beta_{5}*\Delta \gamma^{2}+\beta_{6}*AS^{2}+\varepsilon \end{array}$$

Where ΔE represents either Delta_Positive or Delta_Negative, Δθ= Theta_Change, Δγ=Delta_Gramma, and AS=Affective_Shift. Interaction and quadratic terms account for possible nonlinear saturation and cognitive-affective synergy. To address distributional concerns and small sample size, bootstrapped confidence intervals (*n* = 1,000) were computed for all coefficients (Chaubey et al., 2018).

#### 3.4.3 Nonlinear simulation: feedback-based dynamic modeling

Despite the pre/post structure of the dataset, simulation-based modeling allows for the reconstruction of adaptive emotional adjustment trajectories (Dogan, 2007; Wang and Zhang, 2022). This framework treats predictor variables as strategic input parameters, which adjust iteratively to optimize emotional outcomes. A gradient-based simulation loop was implemented as follows:


$$\Delta \theta_{t+1}=\Delta \theta_{t}+\eta_{1}*\frac{\partial \mathrm{\Delta Ε}}{\partial \Delta \theta }$$

$$\Delta \gamma_{t+1}=\Delta \theta_{t}+\eta_{2}*\frac{\partial \mathrm{\Delta Ε}}{\partial \Delta \gamma }$$

$$AS_{t+1}=AS_{t}+\eta_{3}*\frac{\partial \mathrm{\Delta Ε}}{\partial \mathrm{AS}}$$

Where $\eta_{1}$, $\eta_{2}$, $\eta_{3}$ are learning rates, and the simulation proceeds until ∣ΔΔΕ∣<ε, indicating convergence to a local Nash equilibrium. This framework captures the feedback-driven, co-regulatory emotional adjustment process, emulating how individuals may dynamically recalibrate cognitive load and arousal in response to internal affective cues.

#### 3.4.4 Rationale for model selection

The dual-model structure was designed to address both confirmatory and exploratory aims. Specifically, the linear regression model provides interpretable parameter estimates aligned with predefined hypotheses (H1–H3), offering a theory-driven means of hypothesis testing. In contrast, the nonlinear simulation model enables the exploration of emergent affective trajectories under feedback sensitivity, thereby modeling emotion regulation as an adaptive process. Regularization techniques (e.g., Ridge regression) were deliberately excluded from prioritizing parameter transparency; however, future research may incorporate such methods to improve generalizability beyond the sample.

### 3.5 Tools and techniques

To ensure methodological rigor and computational reproducibility, this study utilized a Python-based analytical pipeline comprising the following libraries:

- Pandas and NumPy: for data preprocessing, score aggregation, and numerical manipulation.
- Statsmodels: for statistical inference and multivariate regression modeling, including bootstrapped CI estimation.
- Seaborn: for creating correlation heatmaps and violin plots.
- Matplotlib: for rendering 3D surfaces of predicted emotional responses across the Theta_Change, Gamma_Change, and Affective_Shift parameter space.
- Scipy (optimize module): for implementing gradient-based simulations under feedback-sensitive conditions.

This toolkit enabled a robust, interpretable, and theoretically grounded modeling pipeline, integrating both descriptive and dynamic components of the proposed emotion regulation framework.

## 4 Results

This section presents empirical findings derived from the linear regression model and the nonlinear simulation, complemented by visualizations and statistical mediation analyses. The results are organized around four central themes: optimal emotional configurations, surface-based dynamic visualizations, mediation mechanisms, and multimodal prediction performance.

### 4.1 Bootstrapped regression results

A multivariate linear regression analysis was performed to assess the predictive influence of neurophysiological and self-reported variables on emotional outcomes, employing bootstrapped confidence intervals (*n* = 1,000) to enhance estimation robustness. Table 3 summarizes the resulting coefficients alongside their 95% confidence intervals.

**Table 3:** Bootstrapped regression coefficients for emotional outcomes (*n* = 1,000).

Variable	Coefficient	95% CI Lower	95% CI Upper
Theta_Change	2.68 × 10^−11^	−2.99 × 10^−12^	7.23 × 10^−11^
Gamma_Change	1.70 × 10^−12^	6.06 × 10^−13^	2.94 × 10^−12^
Delta_Positive	1.69	−0.53	3.90
Delta_Negative	−3.96	−5.84	−2.16
Affective_Shift	5.63	2.74	9.16
Theta × Shift Interaction	1.77 × 10^−10^	−2.60 × 10^−11^	5.04 × 10^−10^
Gamma^2^	1.03 × 10^−23^	2.84 × 10^−24^	2.07 × 10^−23^
Shift^2^	79.43	23.42	152.17

Coefficients reflect unstandardized raw power values; interpretation should be cautious. Future work will apply normalization for comparability.

Results indicate that *affective shift* is the most influential predictor, exerting a substantial and statistically significant positive effect on emotional outcomes with a narrow confidence interval. Gamma_Change showed a statistically significant but small effect, suggesting limited predictive contribution beyond subjective ratings. The significant negative coefficient of Delta_Negative suggests effective downregulation of negative affect. However, the direct effect of *Theta_Change* is not statistically significant; its interaction with *Affective_Shift* points to a context-dependent modulation effect. The nonlinear terms (*Gamma^2^* and *Shift^2^*) also highlight potential saturation or synergistic effects, reinforcing the rationale for a hybrid modeling strategy combining linear and nonlinear elements.

### 4.2 Dynamic evolution of emotional payoff: simulation-based visualization

We implemented a gradient-based nonlinear simulation model to investigate further how emotional outcomes adapt through feedback-driven regulation. This model treats *Theta_Change*, *Gamma_Change*, and *Affective_Shift* as dynamic, co-evolving parameters rather than fixed inputs. By doing so, it transcends conventional pre/post comparisons and constructs a fluid emotional payoff landscape that reflects interactive co-regulation between users and the system. Two main types of visualization outputs were generated to capture the temporal dynamics of the model:

- Payoff surface plots depict emotional returns as functions of varying cognitive load (Theta) and physiological arousal (Gamma) under fixed levels of *Affective_Shift*.
- Optimization trajectory plots illustrate the sequential adaptation of input parameters and their associated emotional payoffs across iterations.

**Figure 2:**
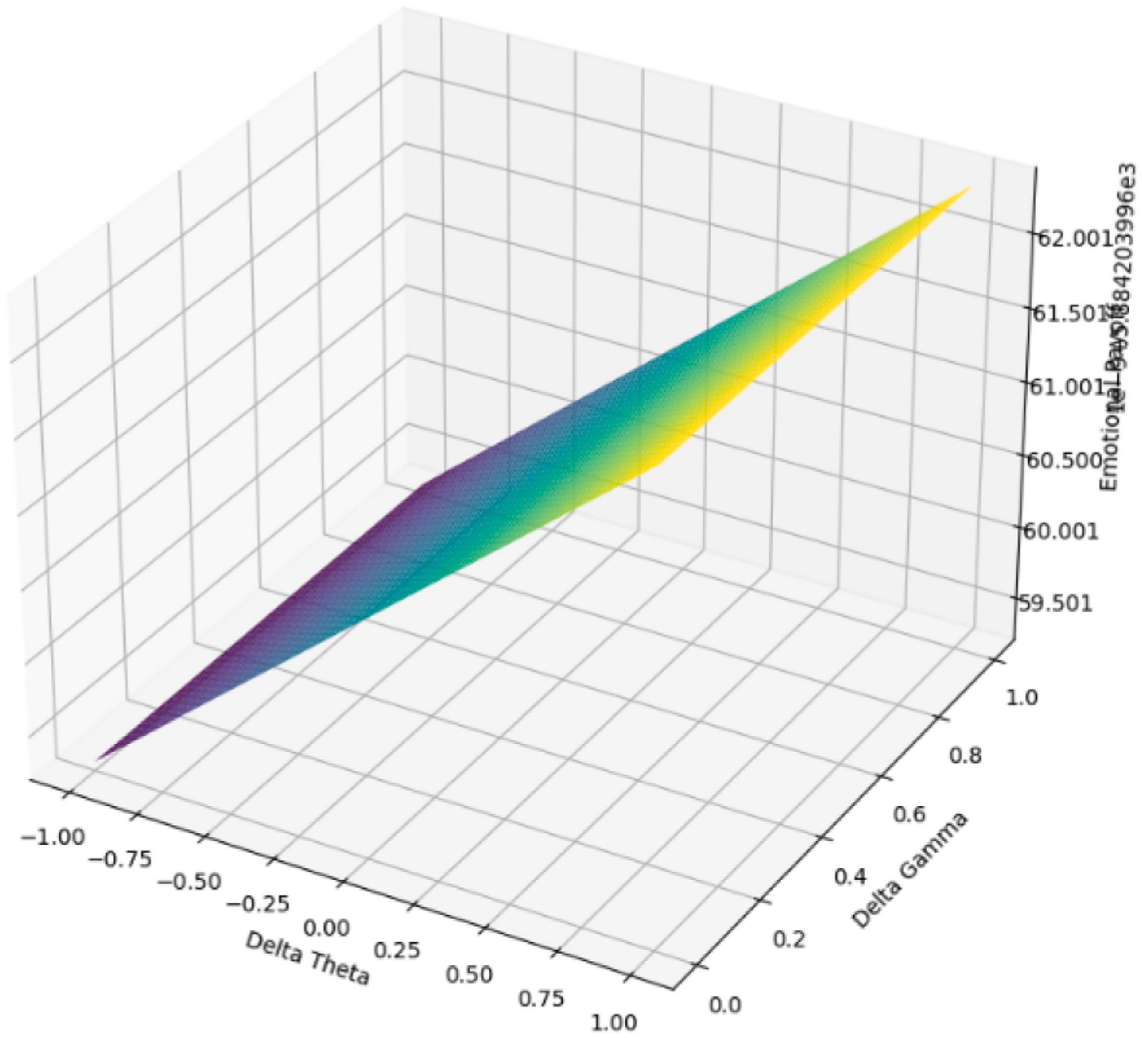
3D payoff surface (affective shift = 10). This is a simulation based on pre/post data, not measured over time.

**Figure 3:**
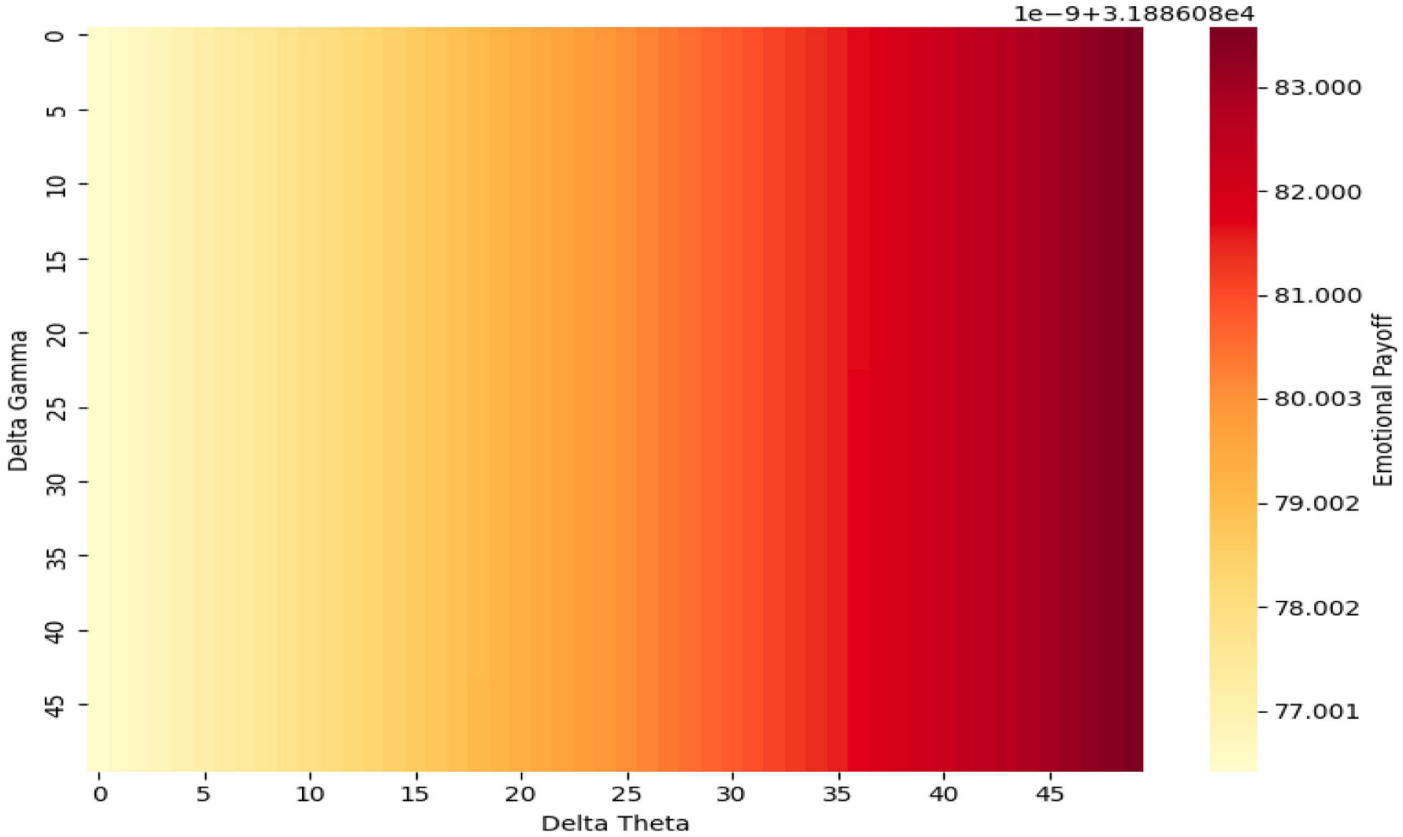
2D heatmap (affective shift = 20). This is a simulation based on pre/post data, not measured over time.

Simulation results theoretically projected that emotional payoff would stabilize within 15–20 iterations, illustrating convergence under the model’s assumptions. This should be interpreted as a conceptual demonstration rather than empirical evidence. Figure 2 presents a 3D payoff surface under moderate affective shift (Affective_Shift = 10). The results indicate that higher Gamma combined with lower Theta levels leads to the most favorable emotional outcomes—supporting a neuro-affective regulation strategy marked by low cognitive effort and high arousal. In contrast, Figure 3 displays a 2D heatmap at a higher affective shift (Shift = 20), revealing a steep emotional gradient that favors high Gamma and low-to-mid Theta configurations, even under more intense affective conditions.

**Figure 4:**
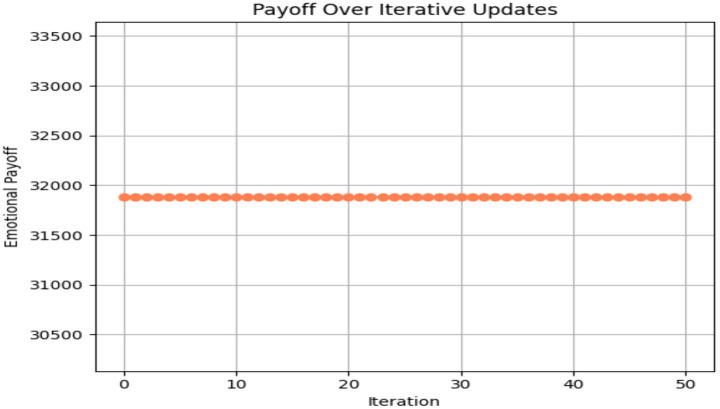
Payoff over across iterations.

**Figure 5:**
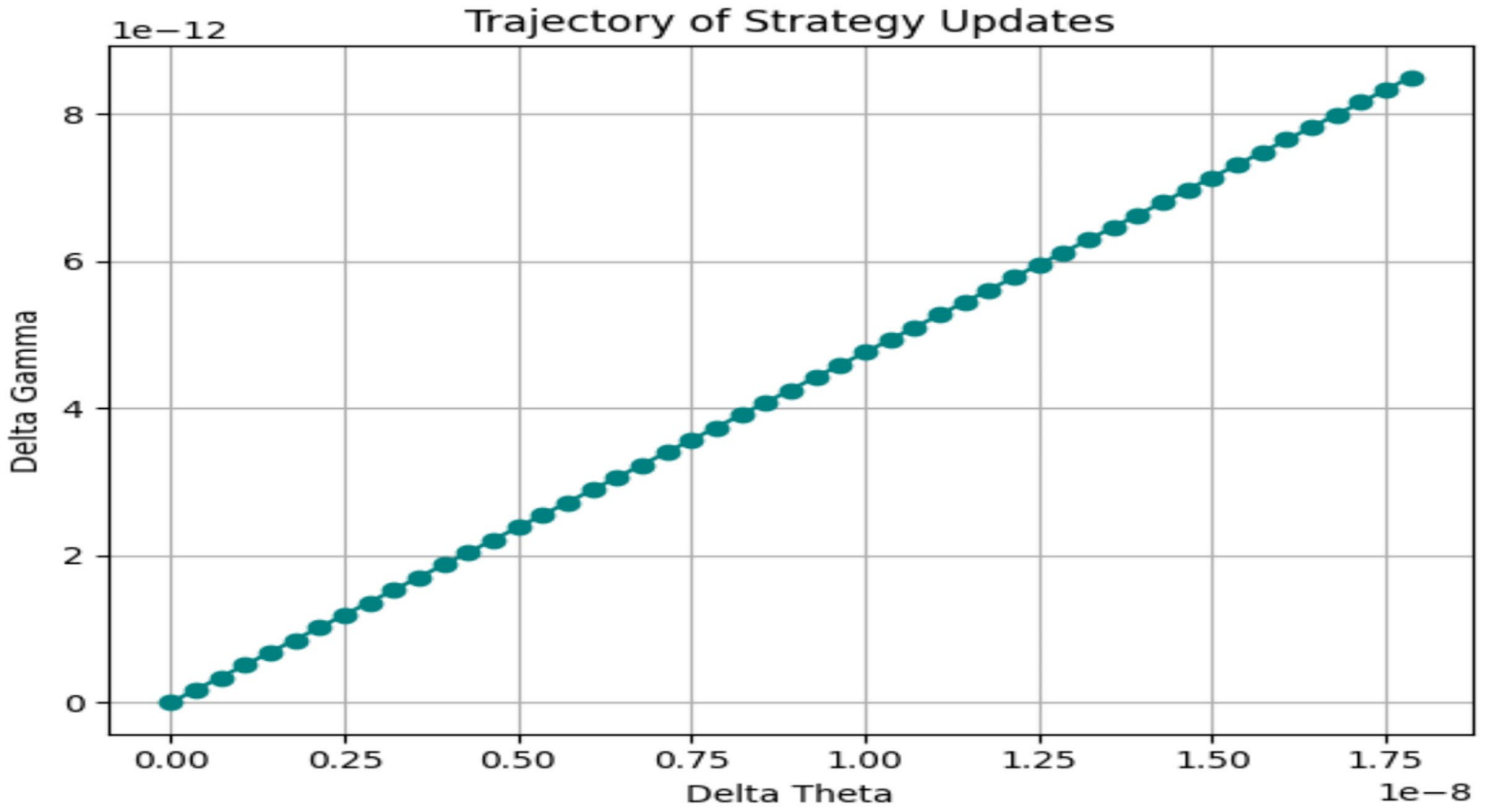
Neuro-affective strategy trajectory.

To illustrate the optimization process over time, Figure 4 depicts the evolution of emotional payoff across 50 simulation steps. A plateau emerges around the 15th iteration, signifying convergence toward a local equilibrium. Meanwhile, Figure 5 traces the joint trajectory of *Theta_Change* and *Gamma_Change*, showing coordinated adjustments that culminate in an optimal neuro-affective configuration. Exploratory mediation models treating Affective_Shift as a mediator did not yield significant indirect effects, consistent with its overlap with the outcome measures.

### 4.3 Multimodal vs. unimodal prediction performance

To assess the predictive contributions of self-report and neurophysiological inputs, three models were evaluated:

- A multimodal model incorporating *Theta_Change*, *Gamma_Change*, and *Affective_Shift*,
- A PANAS-only model using only *Affective_Shift*, and
- An EEG-only model comprising *Theta_Change* and *Gamma_Change*.

As shown in Table 4, the PANAS-only model explained the majority of the variance in Δ_Positive (*R*^2^ = 0.618). The multimodal model that additionally included EEG features showed only a minimal increase in explanatory power (*R*^2^ = 0.637, ΔR^2^ = 0.019), while the EEG-only model accounted for minimal variance (R^2^ = 0.037). These results indicate that subjective self-report measures dominate predictive performance, with EEG contributing only a negligible incremental value in this dataset, thus providing only partial support for H3. The added utility of EEG, therefore, appears limited, highlighting the central role of subjective appraisal in emotion regulation modeling.

**Table 4:** Model performance for predicting Δ_Positive.

Model type	RMSE (mean)	R^2^ (mean)
Multimodal	2.984012823	0.637275554
EEG-only	4.861583567	0.037211339
PANAS-only	3.062875822	0.617849673

## 5 Discussion

### 5.1 Key findings

This study investigated the combined effects of neural activity and subjective affective experience on emotion regulation within the context of interactive art therapy. Grounded in Gross’s emotion regulation theory and Russell’s circumplex model of affect, we formulated and tested three hypotheses using data from electroencephalography (EEG) and PANAS self-reports.

First, we examined whether Theta_Change (representing cognitive load) predicted reductions in negative emotion (Hypothesis 1). The regression analysis revealed that Theta_Change did not reach statistical significance in predicting Δ_Negative (*β* = 2.68 × 10^−11^, 95% CI included zero). However, simulation-based modeling conceptually illustrated potential conditional effects in feedback-sensitive contexts, suggesting a possible nonlinear or threshold-based role. These projections should be viewed as exploratory rather than empirical evidence, offering only tentative support for Hypothesis 1.

Second, we examined whether Gamma_Change (reflecting physiological arousal) predicted increases in positive emotion (Hypothesis 2). The analysis showed that Gamma_Change was a statistically significant predictor of Δ_Positive (β = 1.70 × 10^−12^, 95% CI [6.06 × 10^−13^, 2.94 × 10^−12^]), consistent with its theorized role in emotional enhancement, although the effect size was minimal. This finding provides preliminary rather than strong support for Hypothesis 2.

Third, we evaluated whether a multimodal mode (EEG and PANAS) would outperform unimodal models in predictive accuracy (Hypothesis 3). The multimodal model demonstrated the highest predictive accuracy (*R*^2^ = 0.637), but only slightly higher than the PANAS-only model (*R*^2^ = 0.618), while the EEG-only model accounted for minimal variance (*R*^2^ = 0.037). These findings suggest that PANAS features drive nearly all predictive power, with EEG variables contributing only marginal incremental value. Therefore, Hypothesis 3 received only partial support, as multimodal models performed best but offered negligible improvement over self-report measures alone.

These findings underscore the central role of subjective affective appraisals—particularly Affective_Shift—as robust predictors of emotion regulation outcomes. While EEG-derived features offer mechanistic insights into cognitive load and arousal dynamics, their standalone predictive power remains modest. These results highlight the necessity of a multimodal integration approach, where physiological and self-report measures jointly inform affective modeling in interactive contexts.

### 5.2 Theoretical contributions

According to Gross’s (1998) Process Model, emotion regulation unfolds through antecedent-focused strategies, such as cognitive reappraisal, and response-focused strategies, such as suppression or modulation. In this study, Theta_Change, often associated with frontal-midline theta activity, can be interpreted as a neural marker of cognitive control engaged during reappraisal. While it did not significantly predict emotional outcomes in linear models, its interactive influence with Affective_Shift was conceptually illustrated in dynamic simulations. This projection is consistent with the idea that cognitive regulation may be contextually modulated rather than universally linear—a key tenet of Gross’s theory.

Russell’s Circumplex Model of Affect, which conceptualizes emotion along valence and arousal, further contextualizes the findings. The small positive association between Gamma_Change and Δ_Positive, together with the theoretically projected diminishing returns at higher arousal levels in simulations, aligns with the model’s nonlinear structure. Specifically, moderate to high arousal levels were linked to enhanced emotional outcomes, whereas excessive arousal did not confer additional benefits. This illustrates the notion of bounded optimal emotional zones rather than simple linear increases in affect. The strong influence of Affective_Shift also reflects Russell’s emphasis on subjective appraisal as a central determinant of emotional experience.

In addition, the Emotion Dynamics Theory proposed by Kuppens and Verduyn underscores the temporal and context-sensitive nature of emotional change. This framework helps explain why Affective_Shift consistently outperformed EEG predictors—not merely as a static measurement but as a dynamic indicator of emotional adaptation. Our use of gradient-based simulation to model emotion as a feedback-sensitive process conceptually reflects the orientation of this framework, in which emotional outcomes are emergent phenomena shaped by internal recalibration and real-time interaction with the environment.

These theoretical perspectives converge on a central insight: emotion regulation is a nonlinear, interactive, and multi-level process shaped by the interplay of neural activity, subjective evaluation, and continuous feedback. The present findings reinforce this view, demonstrating that EEG and self-report measures capture complementary aspects of the affective system and that integrative approaches are necessary to understand the full complexity of emotional regulation in immersive, experience-driven settings.

### 5.3 Comparison with existing literature

In line with established theoretical and empirical frameworks (e.g., Kuppens and Verduyn, 2017; Russell, 1980), the present findings reinforce the view that self-reported affective appraisals remain the most robust predictors of emotional change. Specifically, the PANAS-only model yielded the highest explanatory power (*R*^2^ = 0.618), substantially exceeding the predictive capacity of the EEG-only model (*R*^2^ = 0.037). This pattern is consistent with Brans and Verduyn (2014), who contend that subjective appraisal mechanisms are more proximally linked to emotional experience than physiological indices.

While adding EEG-derived features such as Gamma_Change and Theta_Change did not significantly improve overall predictive accuracy, their inclusion offered preliminary mechanistic indications. Notably, Gamma_Change showed a statistically detectable but very minimal effect on Δ_Positive (*β* = 1.70 × 10^−12^, 95% CI [6.06 × 10^−13^, 2.94 × 10^−12^], *p* < 0.001), which is broadly consistent with the theorized role of gamma activity as a neural correlate of affective arousal (Koval et al., 2023; Balconi et al., 2025). This association aligns with prior evidence that links increased gamma-band activity to heightened arousal in emotionally engaging or immersive settings (Balconi and Mazza, 2009; Keil et al., 2001). In contrast, Theta_Change did not reach statistical significance in linear analyses, although its modulatory effects were conceptually illustrated under feedback-sensitive simulations. These findings are in line with the observations of Algarni et al. (2022) and Hosseini et al. (2023), who argue that EEG signals may offer explanatory value—especially in small-sample affective research—even if their standalone predictive utility remains limited.

The current study’s simulation-based modeling approach conceptually aligns with recent work by Merhof and Meiser (2023) and Xu et al. (2020), both of whom demonstrate that dynamic regulatory strategies can be effectively modeled using discretized pre/post designs. However, our framework diverges methodologically from those of Wang and Zhang (2022) and Koval et al. (2023), who emphasized peak Gamma responses for discrete emotion classification. Instead, we foreground the concept of regulatory elasticity, modeling how EEG-based neural indicators dynamically interact with subjective appraisals across iterative simulation steps.

Furthermore, by constructing a feedback-sensitive emotional payoff surface, this study extends prior modeling efforts into the domain of affective neuroscience. This approach enables both descriptive, prescriptive, and predictive insights, illustrating a possible view of emotional regulation as a recursive, self-regulating system sensitive to internal neural fluctuations and external evaluative feedback. While this interpretation stems from simulation rather than continuous-time empirical tracking, the modeled trajectories nonetheless provide a theoretically grounded framework that complements traditional pre-/post-analytic designs.

In contrast to earlier studies that conceptualized emotional change as a static difference between pre- and post-intervention states or as the output of isolated predictors, the present framework integrates subjective and physiological data within a dual-model, feedback-sensitive architecture. This integrated approach facilitates empirical validation through statistical modeling, enabling exploratory insight through dynamic simulation—thereby offering a potentially more comprehensive understanding of emotion regulation in immersive affective environments.

### 5.4 Limitations

Despite offering valuable contributions, this study is subject to several limitations that should be considered when interpreting the findings. First, the relatively small sample size (*n* = 50) limited the statistical power of the analyses and constrained the generalizability of the results. Although bootstrapping procedures (*n* = 1,000) were applied to mitigate sampling variability, future research should endeavor to recruit larger and more heterogeneous samples to enhance external validity. Second, the EEG system employed consisted of only 32 channels, which restricted spatial resolution and reduced the capacity to localize cortical activation sources. Moreover, given that EEG signals contributed only minimally to predictive accuracy in this study, future work may require not only denser arrays but also refined preprocessing and feature extraction strategies to enhance their explanatory value. Employing high-density EEG arrays or combining EEG with complementary neuroimaging modalities such as functional near-infrared spectroscopy (fNIRS) could offer more nuanced insights into brain–emotion relationships. Third, while widely used in affective neuroscience, the pre/post experimental design was limited in its ability to capture the dynamic, moment-to-moment fluctuations of emotional states. Although the simulation-based framework offered an exploratory illustration of regulation over time, empirical tracking of real-time changes with higher temporal granularity is required to examine whether such projected dynamics hold in practice. Fourth, emotional states were measured exclusively using the PANAS self-report scale, which may introduce memory biases and be susceptible to social desirability effects. Future studies should combine PANAS with continuous or passive emotion-tracking methodologies—such as ecological momentary assessment (EMA), physiological signals (e.g., GSR, HRV), or computer vision–based facial expression analysis—to enhance ecological validity and response fidelity.

### 5.5 Future research directions

To build upon these limitations, several directions for future research and design innovation can be proposed to develop further and extend the present framework. The current findings emphasize the importance of integrating EEG-derived neural indicators with PANAS-based self-report data to model emotion regulation as a multimodal, adaptive process governed by feedback sensitivity. Specifically, future research could further examine whether emotional improvement is associated with patterns such as reduced cognitive load (Theta_Change), moderate physiological arousal (Gamma_Change), and substantial subjective reappraisal (Affective_Shift).

Future research should move toward real-time, high-resolution tracking of emotional responses, drawing on multimodal data streams (e.g., EEG, galvanic skin response, heart rate variability, eye-tracking), which have been shown to be effective in emotion prediction and adaptive systems (Jiang et al., 2025). This would enable more granular modeling of affective dynamics and support the construction of closed-loop systems capable of real-time adaptation to user feedback. Personalization is another essential priority. Integrating user-specific factors—such as personality traits, baseline affective profiles, and contextual influences—can increase the robustness and relevance of the model across diverse populations, including older adults, neurodivergent individuals, and participants in culturally varied or artistically distinct environments.

From a design standpoint, emotion regulation can be approached as a malleable design parameter. Elements such as spatial configuration, interaction pacing, and multisensory ambient features (e.g., soundscapes, lighting, haptics) can be purposefully tailored to guide users toward emotional equilibrium or expressive catharsis. Given the observed nonlinearities in affective trajectories, Future modeling efforts could benefit from exploring advanced sequence learning approaches—such as LSTM or attention-based models—which have shown promise in capturing multimodal and time-varying affective signals in interactive contexts (Wöllmer et al., 2013; Ding, 2023).

In sum, embedding interpretable emotional models within responsive environments offers a promising trajectory for affect-aware, personalized, and inclusive interactive systems—with potential applications ranging from therapeutic interventions to creative and educational settings. Based on these insights and the current findings, Future studies could test whether decreases in cognitive load (Theta_Change) under real-time feedback are linked to positive affect, whether moderate increases in Gamma_Change predict more effective regulation when paired with high subjective reappraisal, and whether personalized affective models incorporating user-specific traits outperform generalized models.

## 6 Conclusion

This study proposed and tested a dual-model framework for characterizing emotional change within interactive art therapy, integrating EEG-derived neural signals and PANAS-based self-report data under a feedback-sensitive architecture. Among the predictors examined, subjective affective shift emerged as the most robust indicator of regulatory success, while simulation results conceptually illustrated potential nonlinear co-regulatory patterns involving cognitive (Theta_Change) and physiological (Gamma_Change) components. These findings offer preliminary alignment with multidimensional theories of emotion regulation and practical guidance for designing effectively intelligent systems. Future work should pursue higher temporal resolution, deeper personalization strategies, and greater integration of real-time affective computing techniques, which could contribute to the advancement of emotionally adaptive technologies in immersive, human-centered contexts.

## Data Availability

The dataset analyzed in this study is publicly available via the Science Data Bank and can be accessed at: https://cstr.cn/31253.11.sciencedb.08758, The computational code supporting the findings of this study is available from the first author upon reasonable request.

## References

[ref1] Algarni M. Saeed F. Al-Hadhrami T. Ghabban F. Al-Sarem M. (2022). Deep learning-based approach for emotion recognition using electroencephalography (EEG) signals using bi-directional long short-term memory (bi-LSTM). Sensors 22:2976. doi: 10.3390/s22082976, PMID: 35458962 PMC9033053

[ref2] Balconi M. Angioletti L. Allegretta R. A. (2025). Which type of feedback—positive or negative- reinforces decision recall? An EEG study. Front. Syst. Neurosci. 18:1524475. doi: 10.3389/fnsys.2024.1524475, PMID: 39845868 PMC11751025

[ref3] Balconi M. Mazza G. (2009). Brain oscillations and BIS/BAS (behavioral inhibition/activation system) effects on processing masked emotional cues.: ERS/ERD and coherence measures of alpha band. Int. J. Psychophysiol. 74, 158–165. doi: 10.1016/j.ijpsycho.2009.08.006, PMID: 19709636

[ref4] Barnett K. S. Vasiu F. (2024). How the arts heal: a review of the neural mechanisms behind the therapeutic effects of creative arts on mental and physical health. Front. Behav. Neurosci. 18. doi: 10.3389/fnbeh.2024.1422361, PMID: 39416439 PMC11480958

[ref5] Belkofer C. M. Van Hecke A. V. Konopka L. M. (2014). Effects of drawing on alpha activity: a quantitative EEG study with implications for art therapy. Art Ther. 31, 61–68. doi: 10.1080/07421656.2014.903821

[ref6] Brans K. Verduyn P. (2014). Intensity and duration of negative emotions: comparing the role of appraisals and regulation strategies. PLoS One 9:e92410. doi: 10.1371/journal.pone.0092410, PMID: 24670979 PMC3966809

[ref7] Chaubey Y. P. Khurana M. Chandra S. (2018). Confidence intervals based on resampling methods using ridge estimator in linear regression model. New Trends Math. Sci. 4, 77–86. doi: 10.20852/ntmsci.2018.318

[ref8] Chenghong Cen Kang Li Jing Xu Guanghui Huang Tan Jiang. Harnessing new Frontiers in art therapy: An interactive installation integrating EEG, VR, and AIGC for the alleviation of negative emotions[DS/OL]. V1. Science Data Bank, (2024). Available online at: https://cstr.cn/31253.11.sciencedb.08758.

[ref9] Ding Y. (2023). Neurophysiology-inspired neural networks for affective brain-computer interfaces. Singapore: Nanyang Technological University.

[ref10] Dogan G. (2007). Bootstrapping for confidence interval estimation and hypothesis testing for parameters of system dynamics models. Syst. Dyn. Rev. 23, 415–436. doi: 10.1002/SDR.362

[ref9005] Dubey M. Chitturi V. Tayade P. Sharma R. Kaur S. (2024). EEG Cortical Sources of Theta, Beta and Gamma Frequencies Orchestrate Audio-Visual Interactions. J. Clinical Med. 14:1895. doi: 10.3390/jcm14061895 PMC11561918 39554719

[ref9006] Gross J. J. (1998). The emerging field of emotion regulation: An integrative review. Rev. Gen. Psychol. 2, 271–299. doi: 10.1037/1089-2680.2.3.271

[ref11] Gross J. J. (2015). Emotion regulation: current status and future prospects. Psychol. Inq. 26, 1–26. doi: 10.1080/1047840X.2014.940781

[ref12] Harmon-Jones E. Gable P. A. (2018). On the role of asymmetric frontal cortical activity in approach and withdrawal motivation: an updated review of the evidence. Psychophysiology 55:e12879. doi: 10.1111/psyp.12879, PMID: 28459501

[ref13] Hinz L. D. (2020). Expressive therapies continuum: a framework for using art in therapy. London: Routledge.

[ref14] Hosseini M. S. K. Firoozabadi S. M. Badie K. Azadfallah P. (2023). Personality-based emotion recognition using EEG signals with a CNN-LSTM network. Brain Sci. 13:947. doi: 10.3390/brainsci13060947, PMID: 37371425 PMC10296308

[ref15] Jiang D. Jin D. Zhuang J. Tan D. Chen D. Liang Y. (2021). A computational model of emotion model based on audio-visual stimuli understanding audiovisual stimuli and personalized regulation with concurrency. Concurrency Computat. Pract. Exper. 33:e6269. doi: 10.1002/cpe.6269

[ref21] Jiang X. Wang Y. Chen Z. (2025). Neuro-Feedback VR: Real-Time Emotion Manipulation and Adaptive Environments for Enhanced Performance and Therapy. Proceedings of the IEEE Conference on Cognitive and Computational Environments, 1–8. doi: 10.1109/CE2CT64011.2025.10939880

[ref16] Kaimal G. Carroll-Haskins K. Mensinger J. L. Dieterich-Hartwell R. Biondo J. Levin W. P. (2020). Outcomes of therapeutic artmaking in patients undergoing radiation oncology treatment: a mixed-methods pilot study. Integr. Cancer Ther. 19:1534735420912835. doi: 10.1177/1534735420912835, PMID: 32316856 PMC7177989

[ref17] Keil A. Müller M. M. Gruber T. Wienbruch C. Stolarova M. Elbert T. (2001). Effects of emotional arousal in the cerebral hemispheres: a study of oscillatory brain activity and event-related potentials. Clin. Neurophysiol. 112, 2057–2068. doi: 10.1016/s1388-2457(01)00654-x, PMID: 11682344

[ref18] Koval P. Kalokerinos E. K. Greenaway K. H. Medland H. Kuppens P. Nezlek J. B. . (2023). Emotion regulation in everyday life: mapping global self-reports to daily processes. Emotion 23:357. doi: 10.1037/emo0001097, PMID: 35588386

[ref19] Kuppens P. Verduyn P. (2017). Emotion dynamics. Curr. Opin. Psychol. 17, 22–26. doi: 10.1016/j.copsyc.2017.06.004 28950968

[ref20] Lahijanian M. Aghajan H. (2023). 40Hz auditory entrainment promotes synchronization between frontal and parietal regions of the brain. In 2023 31st international conference on electrical engineering (ICEE) (pp. 772–775)

[ref22] Lusebrink V. B. Hinz L. D. (2020). Cognitive and symbolic aspects of art therapy and similarities with large-scale brain networks. Art Ther. 37, 113–122. doi: 10.1080/07421656.2019.1691869

[ref23] Merhof V. Meiser T. (2023). Dynamic response strategies: accounting for response process heterogeneity in IRTree decision nodes. Psychometrika 88, 1354–1380. doi: 10.1007/s11336-023-09901-0, PMID: 36746887 PMC10656330

[ref24] Russell J. A. (1980). A circumplex model of affect. J. Pers. Soc. Psychol. 39, 1161–1178. doi: 10.1037/h0077714

[ref25] Schauberger G. Tutz G. (2023). catdata: Categorical Data (Version 1.2.3) [R package]. Available online at: https://CRAN.R-project.org/package=catdata

[ref26] Slovak P. Antle A. Theofanopoulou N. Daudén Roquet C. Gross J. Isbister K. (2023). Designing for emotion regulation interventions: an agenda for HCI theory and research. ACM Trans. Comput.-Hum. Interact. 30, 1–51. doi: 10.1145/3569898

[ref27] Southward M. W. Sauer-Zavala S. Cheavens J. S. (2021). Specifying the mechanisms and targets of emotion regulation: a translational framework from affective science to psychological treatment. Clin. Psychol. Sci. Pract. 28:168. doi: 10.1037/cps0000003, PMID: 40991787

[ref28] Stuckey H. L. Nobel J. (2010). The connection between art, healing, and public health: a review of current literature. Am. J. Public Health 100, 254–263. doi: 10.2105/AJPH.2008.156497, PMID: 20019311 PMC2804629

[ref9004] Tang Z. Li X. Xia D. Hu Y. Zhang L. Ding J. (2022). An art therapy evaluation method based on emotion recognition using EEG deep temporal features. Multimedia Tools and Applica. 81, 7085–7101. doi: 10.1007/s11042-022-12002-2

[ref29] Wang Q. Liu F. Yang B. Yang Z. Huang J. Xu Y. . (2025). An EEG-based positive feedback mechanism for VR mindfulness meditation to improve emotion regulation. IEEE Trans. Comput. Soc. Syst. 99, 1–13. doi: 10.1109/TCSS.2025.35577112025

[ref30] Wang X. Zhang Y. (2022). Simulation of adaptive emotional regulation using discrete pre-post designs. In Proceedings of the 2022 International conference on affective computing (AC).

[ref31] Wöllmer M. Kaiser M. Eyben F. Schuller B. Rigoll G. (2013). LSTM-modeling of continuous emotions in an audiovisual affect recognition framework. Image Vis. Comput. 31, 153–163. doi: 10.1016/j.imavis.2012.03.001

[ref32] Xu H. Wu S. Cai H. Ai K. Xu M. (2020). Modeling and simulation of dynamic emotion diffusion in public agendas. In 2020 IEEE international conference on systems, man, and cybernetics (SMC) (pp. 58–63)

